# Post-traumatic Stress Disorder Symptoms in COVID-19 Survivors 6 Months After Hospital Discharge: An Application of the Conservation of Resource Theory

**DOI:** 10.3389/fpsyt.2021.773106

**Published:** 2022-01-04

**Authors:** Bingyi Wang, Xue Yang, Leiwen Fu, Yuqing Hu, Dan Luo, Xin Xiao, Niu Ju, Weiran Zheng, Hui Xu, Yuan Fang, Paul Shing Fong Chan, Zhijie Xu, Ping Chen, Jiaoling He, Hongqiong Zhu, Huiwen Tang, Dixi Huang, Zhongsi Hong, Xiaojun Ma, Yanrong Hao, Lianying Cai, Jianrong Yang, Shupei Ye, Jianhui Yuan, Yao-Qing Chen, Fei Xiao, Zixin Wang, Huachun Zou

**Affiliations:** ^1^School of Public Health (Shenzhen), Sun Yat-sen University, Shenzhen, China; ^2^The Jockey Club School of Public Health and Primary Care, Faculty of Medicine, The Chinese University of Hong Kong, Hong Kong, Hong Kong SAR, China; ^3^Center for Optometry and Visual Science, The People's Hospital of Guangxi Zhuang Autonomous Region, Nanning, China; ^4^Department of Early Childhood Education, The Education University of Hong Kong, Hong Kong, Hong Kong SAR, China; ^5^The Fifth Affiliated Hospital of Sun Yat-sen University, Zhuhai, China; ^6^Guangdong Provincial People's Hospital, Guangzhou, China; ^7^Department of Scientific Research, The People's Hospital of Guangxi Zhuang Autonomous Region, Nanning, China; ^8^Department of Education, The People's Hospital of Guangxi Zhuang Autonomous Region, Nanning, China; ^9^Department of Hepatobiliary, Pancreas and Spleen Surgery, The People's Hospital of Guangxi Zhuang Autonomous Region, Nanning, China; ^10^Department of Emergency, SSL Central Hospital of Dongguan City, Dongguan, China; ^11^Shenzhen Nanshan District Center for Disease Control and Prevention, Shenzhen, China; ^12^School of Public Health, Shanghai Jiao Tong University School of Medicine, Shanghai, China; ^13^Shenzhen Center for Disease Control and Prevention, Shenzhen, China; ^14^Kirby Institute, University of New South Wales, Sydney, NSW, Australia

**Keywords:** post-traumatic stress disorder (PTSD), hospitalization-related factors, resource loss and gain, resilience, conservation of resource theory (COR)

## Abstract

COVID-19 survivors who had acute respiratory symptoms might experience prolonged post-traumatic stress disorder (PTSD) due to further rehabilitation, somatic symptoms and related distress. The conservation of resource (COR) theory is a well-developed theory to understand how people develop PTSD symptoms in traumatic events. The current study aimed to examine the potential factors of PTSD symptoms and interrelationships among this factors among COVID-19 survivors based on the COR theory. This cross-sectional telephone survey enrolled 199 COVID-19 patients (Mean age = 42.7; 53.3% females) 6 months after their hospital discharge in five Chinese cities (i.e., Wuhan, Shenzhen, Zhuhai, Dongguan, and Nanning). The results showed that 7% of participants were classified as having probable PTSD. The significant potential factors relating to PTSD symptoms included socio-demographic status, hospitalization experiences, post-hospitalization experiences, and psychological status. Besides, the proposed statistical mediation model based on the COR framework showed good model fit, χ^2^(df) = 17.286 (5), *p* = 0.004, CFI = 0.962, NNFI = 0.951, RMSEA = 0.077. Perceived resource loss/gain fully mediated the association between exposure to other patients' suffering during hospitalization and PTSD symptoms, and partially mediated the relationships from somatic symptoms/perceived impact of being infected with COVID-19 after discharge to PTSD symptoms. On the other hand, resilience was a full mediator in the relationship from ICU experience to PTSD symptoms and a partial mediator in the relationship from perceived impact to PTSD symptoms. The results provide preliminary support on applying the COR theory to understand the factors of PTSD symptoms among COVID-19 survivors. Interventions to reduce PTSD symptoms in this population can be developed based on the modifiable psychosocial mediators.

## Introduction

Post-traumatic stress disorder (PTSD) is defined as the development of symptoms related to intrusion, avoidance, negative alterations in cognition and mood, and arousal and reactivity following exposure to a traumatic event (1). Post-traumatic stress symptoms (PTSS) have also been associated with functional impairment among individuals who do not meet full diagnostic criteria (2). Based on information garnered from human coronavirus outbreaks in the past, specifically SARS and MERS, PTSD and PTSS may become a significant health concern among COVID-19 survivors (3, 4). To our knowledge, only five studies on PTSD among COVID-19 survivors have been reported (sample size range: 126–675); the prevalence of PTSD ranged from 12.4 to 28% (5–9). For example, a Chinese study reported that among 675 survivors, 12.4% were diagnosed with PTSD (5.2% had been in intensive care-ICU; 14.2% had been given substantial doses of corticosteroid during hospital treatment; 2.4% had received invasive mechanical ventilation) (7). The prevalence suggests that health care providers should be prepared to evaluate the associated risks and make recommendations in response to the increase of PTSD and PTSS among COVID-19 survivors.

### Potential Causes of PTSD in COVID-19 Survivors

Severity of symptoms, treatment-related negative experiences and pain, and collective trauma during hospitalization are potential causes of PTSD in COVID-19 survivors (10). Patients with COVID-19 might experience respiratory symptoms and respiratory failure (11), such experiences create extreme stressors for patients, including fear of death, pain from medical interventions, limited ability to communicate, and feelings of loss of control (10). Invasive ventilation and longer duration of mechanical ventilation could cause post-traumatic stress to ICU treatment survivors (12–15). Such treatments have been associated with an increased risk for PTSS (16, 17) and associated PTSD prevalence was estimated to range from 14 to 51% (18). Patients who required mechanical ventilation at ICU also reported symptoms including feelings of guilt, mood swings, sleep disturbance, and memories of panic and suffocation (19). COVID-19 survivors who had acute respiratory symptoms might experience prolonged PTSD due to further rehabilitation, somatic symptoms and related distress (20). Additionally, COVID-19 survivors might experience collective trauma during hospitalization from witnessing other patients' suffering or death, potentially causing PTSD (7). Existing literature on these factors is rare and reported inconsistent results. A study in Italian reported that one out of five patients hospitalized for COVID-19 was diagnosed with PTSD or subthreshold PTSD at 3-month follow-up (9). However, negative correlations between hospitalization experiences and PTSD, insignificant correlations between residual clinical damage and PTSD (8), and negative correlations between duration of hospitalization and PTSD (6) were reported in other cultures. The inconsistent results highlight the necessity of more empirical studies to understand these relationships between hospitalization/post-hospitalization experiences and PTSD and their underlying mechanisms.

### Potential Mediators and Framework of PTSD

Resources can be referred to anything that a person values, such as objects (e.g., house and phone), conditions (e.g., stable employment and good health), personal characteristics, (e.g., optimism and hope), energies (e.g., knowledge), and social resources (e.g., interpersonal relationships) (21). Trauma-related experiences may reduce COVID-19 survivors' core resources that support their well-being and help them to cope with stress during the epidemic and afterwards. For example, severe symptoms of COVID-19 and comorbid conditions such as hypertension, coronary artery disease and stroke, and diabetes may increase one's perception of personal resource loss, such as deteriorated physical and emotional health status, sleep quality, quality of life, and working capacity and opportunity (22). Limited face-to-face interactions with others during and after treatment due to concern of infection may reduce perceived social resources (e.g., reduced social network, social connection, and social support, and increased social stigma) among patients with COVID-19 and survivors. Experiencing pain and witnessing others patients' suffering during treatment and hospitalization may damage their sense of mastery, self-efficacy, and resilience (18, 23). In turn, such perceived losses may explain why and how these COVID-19 survivors develop and maintain PTSD after recovery or discharge from hospitals. Based on the Conservation of Resource (COR) theory, the mediation role of “loss of resources” may explain the link between traumatic events and PTSD (24). This theory was originally developed to understand the processes of experiencing, coping with, and overcoming chronic and traumatic stress, and how and why people develop PTSD (24). According to the theory, losing core resources, including both personal and social resources due to traumatic events (e.g., health-related crises) and related negative experiences can prolong PTSD, while gaining such resources can facilitate recovery from PTSD (25). Personal, physical and psychological resources are internal resources that can be possessed and mobilized by the self (e.g., psychological resilience, self-efficacy, sense of control over one's life, and optimism) (26). Social resources refer to external resources that are embedded within the physical environment and interpersonal interactions (27). This theory emphasizes that accelerated loss of resources, particularly those that are most valued by the individual, can lead to traumatic stress. Furthermore, it postulates that regarding PTSD, loss of resources has greater impact than gain because individuals tend to strive to protect themselves from resource loss. The COR theory has been widely used to explain the development of PTSD and psychological distress in the contexts of natural disasters (28, 29), diseases (30), and socio-political movements (31). We, however, did not identify any study which have applied this theory in explaining the development of PTSD in COVID-19 survivors.

### The Present Study

The study aimed to investigate the prevalence and potential factors relating to PTSD in COVID-19 survivors. We were particularly interested in factors related to the experiences and perceptions during and after treatment and hospitalization (e.g., the severity of COVID-19 symptoms, perceived impact of COVID-19, somatic symptoms after hospitalization, and witnessing other patients' suffering during hospitalization) (10, 20, 32). Furthermore, this study applied the COR framework and tested a statistical mediation model in which negative experiences and perceptions related to COVID-19 and hospitalization (e.g., the severity of COVID-19 symptoms, perceived impact of COVID-19, somatic symptoms after hospitalization, and witnessing other patients' suffering during hospitalization) would be positively associated with resource loss (e.g., personal and social resources and psychological resilience) and in turn increase the risk of PTSD in COVID-19 survivors. It is hypothesized that (1) negative experiences and perceptions during and after treatment and hospitalization would be positively associated with PTSD; and (2) these associations would be mediated by perceived loss of resources (e.g., reduced personal and social resources and psychological resilience).

## Methods

### Study Design and Data Collection

This is a cross-sectional telephone survey among 199 COVID-19 patients, conducted 6 months after their hospital discharge. Our participants were: (1) patients who were diagnosed with COVID-19 and hospitalized, regardless of severity level at admission, or duration of stay, ICU admission or treatment received during hospitalization; (2) recovered from acute infection by COVID-19 and were discharged; (3) least 18 years old, and (4) confirmed to have given informed consent to participate in the survey.

The study was conducted between August and September 2020. Two-stage cluster sampling was used, with five hospitals being conveniently selected and all the survivors discharged from the hospitals between February 1 and April 30 2020 were invited. Specifically, the conveniently selected study sites included five hospitals located in five Chinese cities (i.e., Wuhan, Shenzhen, Zhuhai, Dongguan, and Nanning,). Wuhan is the capital city of Hubei Province which is the most heavily affected city by the COVID-19 epidemic in China. Shenzhen, Zhuhai, and Dongguan are cities in Guangdong Province; this Province has the second largest number of confirmed COVID-19 cases in China. Nanning is the capital city of Guangxi Province, which is relatively less affected by COVID-19 epidemic. According to the treatment guidelines in China, COVID-19 patients discharged from hospitals are required to quarantine at centralized facilities for 14 days, followed by an additional 14-day home quarantine. Hospitals will keep their contact information for follow-up assessments and services after discharge. Thus, staffs from the five participating hospitals facilitated the recruitment process for this study. Hospital staffs contacted all the COVID-19 survivors discharged from the hospitals and screened for eligible participants under verbal consent. Eligible participants were briefed about the purpose and logistics of the study and were invited to attend a 30–40-min telephone interview, conducted by trained interviewers (33). Telephone interviews were arranged on an appointment basis (33). Informed consent was obtained from each participant before the interview. Participants were assured that identifiable information would be kept confidential and they are free to withdraw without affecting their access to other medical services. No incentives were given to the participants. Ethics approval was obtained from the Sun Yat-sen University (Shenzhen) (Ref#2020-031).

### Participants

Among 317 discharged COVID-19 patients from the five hospitals, 27 were excluded for being under 18 years old, 22 were unreachable due to change of telephone number and one was deceased in a car accident. The research team contacted the remaining 267 eligible patients and 68 refused to participate due to lack of time. One hundred and ninety-nine eligible participants provided consent and completed the telephone survey. The average response rate was 74.5% (Wuhan: 31/49, 63.3%; Shenzhen: 38/50, 76.0%; Zhuhai: 39/51, 76.5%; Dongguan: 35/45, 77.8%; and Nanning: 56/72, 77.8%).

### Measures

A panel consisting of one epidemiologist, two public health researchers, a health psychologist, and a clinician was formed to develop the questionnaire used in the current study.

### Independent Variables

#### During Hospitalization

Exposure to/witnessing of other patients' suffering during hospitalization was measured by two questions (i.e., “Did you witness other patients suffering from pain due to COVID-19 symptoms during hospitalization” and “Did you witness death of other patients with COVID-19 during hospitalization”). Participants answered with “Yes” or “No” to the questions.

Information about severity level of COVID-19 at hospital admission, days in the hospital, ICU admission, use of invasive ventilation and corticosteroid therapy, and presence of severe complications of COVID-19 were extracted from their medical record.

#### After Discharge

Somatic symptoms after discharge were measured by the Patient Health Questionnaire (PHQ-15) (34). PHQ-15 is a somatic symptom subscale derived from the full PHQ. It inquires about 15 somatic symptoms or symptom clusters that account for more than 90% of the physical complaints (excluding upper respiratory tract symptoms) reported in the outpatient setting (34). The symptoms inquired in the PHQ-15 include 14 of the 15 most prevalent DSM-IV somatization disorder somatic symptoms (i.e., those with a prevalence of 3% or greater in the general population) (35). Participants rated the severity of each symptom as 0 (“not bothered at all”), 1 (“bothered a little”), or 2 (“bothered a lot”). A higher total score indicates a greater somatic symptom severity. The internal consistency of the scale was acceptable in the current sample (Cronbach's alpha = 0.88).

Perceived impact of being infected with COVID-19 was assessed by three questions constructed by the research team. Participants were asked to what extent do they think COVID-19 infection has adverse impacts on their life, work, and socializing, respectively. Questions were rated on Likert scales, ranging from 0 (no influence at all) to 10 (severe influence). A higher score indicates a greater negative influence of COVID-19 infection on the survivors. The reliability of the three questions was good (Cronbach's alpha = 0.89).

#### Mediators

Resource loss/gain was measured by eight questions that were constructed to assess loss or gain of personal resources (i.e., activities of daily living, working capacity, financial status, quality of life, emotional status, and sleep quality) and social resources (i.e., relationships with family and friends). The items were derived from a comprehensive literature review (25, 36) and created by the research team that includes psychologists and epidemiologists. The items were rated by using three-point Likert scales (1 = loss, 2 = no change, 3 = gain). A lower score indicates a tendency of perceived resource loss as a result of COVID-19 infection, while a higher score indicates a tendency of perceived resource gain. The Cronbach's alpha of the scale was relatively low but marginally acceptable (Cronbach's alpha = 0.66).

Resilience was measured by the 2-item Connor-Davidson Resilience Scale (CD-RISC2). The two items (“Able to adapt to change” and “Tend to bounce back after illness or hardship”) were rated on Likert scales, ranging from 1 (strongly disagree) to 5 (strongly agree). A higher total score indicates a higher level of psychological resilience. The Cronbach's alpha of the scale was 0.90 in the current sample.

#### Outcome

PTSD symptoms due to being infected with COVID-19 were measured by the 8-item Post-Traumatic Stress Disorder scale (PTSD-8) (37). The items correspond to the DSM-IV criteria for PTSD to measure three core symptoms of PTSD, including intrusion, avoidance, and hypervigilance. The measure is short and feasible for telephone surveys. The items were answered on a Likert scale, ranging from 1 (not at all) to 5 (all the time). The summed score of the eight items' scores provides a score for symptom severity. Probable PTSD was defined using a threshold of at least one symptom from each PTSD subscale with an item score that was ≥3 (37). Many studies used the summed score of PTSD-8 as a continuous variable with the score ranging from 8 to 40 (38–41). The Chinese version has been used in previous studies (42, 43). The internal consistency as measured by Cronbach's alpha (0.89) was good in the current sample.

### Data Analyses

The percentage of missing data was low (<5%) and they were replaced by multiple imputation. Descriptive statistics were computed for both background and psychological variables. Simple linear regression analyses involving the analysis of a single independent variable were conducted to identify the potential significant background or psychological factors of PTSD symptoms. Standardized regression coefficients (β) and 95% of their confidence intervals (CIs) were reported. The significant variables were further included in path analyses. Path analyses with maximum likelihood estimation approach were conducted to test the proposed statistical mediation models of PTSD symptoms. The Comparative Fit Index (CFI) > 0.90, the NNFI (Non-Normed Fit Index) > 0.90 and the Root Means Square Error of Approximation (RMSEA) < 0.08 suggest acceptable model fit. Bootstrapping based on 5,000 bootstrap samples was performed to test indirect effects. A statistically significant indirect effect would be observed when the CI did not include zero. The level of statistical significance was 0.05. SPSS version 21.0 and AMOS were used. This was a secondary analysis of a study investigating the behaviors and mental health of COVID-19 survivors in China.

## Results

As Table 1 showed, the mean age of the participants was 42.7 years. Over half of the participants were female (53.3%), were married or cohabited with a partner (81.9%), did not have permanent residency in the city (73.4%), had personal income less than RMB 6,000 (USD 850) per month (74.4%), had a full-time work (59.8%), and had children (80.4%, *n* = 160). 43.2% of the participants had college education or above. During hospitalization, most of the participants did not have ICU, invasive assisted ventilation, hormone therapy, serious complications, or sequelae of COVID-19. Half of the participants were classified as having common symptoms of COVID-19. The average length of stay at the hospital was 20.9 days. Of the participants, 7% were classified as having probable PTSD (*n* = 14). The continuous variable of PTSD would be used in the following analyses.

**Table 1:** Background characteristics, physical status and psychosocial status of the participants (*n* = 199).

	***n***	**%**	**Mean**	**SD**
**Background characteristics**				
Age group (years)			42.723	17.528
18–30	33	16.6		
31–40	59	29.6		
41–50	35	17.6		
51–60	33	16.6		
>60	39	19.6		
Sex				
Male	93	46.7		
Female	106	53.3		
Relationship status				
Currently single	36	18.1		
Married/cohabited with a partner	163	81.9		
Having children				
No	39	19.6		
Yes	160	80.4		
Highest education attained				
Middle school or below	53	26.6		
High school	57	28.7		
College and above	86	43.2		
Refuse to disclose	3	1.5		
Permanent residents of the city				
No	146	73.4		
Yes	53	26.6		
Monthly personal income (¥)				
No fixed income	71	35.7		
<3,000	25	12.6		
3,000–5,999	52	26.1		
6,000–9,000	24	12.1		
≥10,000	27	13.6		
Employment status				
Full-time employment	80	40.2		
Free-lanced	31	16.1		
Students	15	7.5		
Unemployed	17	8.5		
Retired	55	27.6		
**Experiences during hospitalization**				
Clinical classification of COVID-19 at entry				
Asymptomatic	3	1.5		
Mild	42	21.1		
Common	111	55.8		
Severe	25	12.6		
Critically severe	18	9.0		
ICU experience (No)	194	97.5		
Invasive assisted ventilation (No)	192	96.5		
Hormone therapy (No)	175	87.9		
Serious complications (No)	188	94.5		
Length of stay (days)			20.883	15.831
Sequelae of COVID-19 before discharge (No)	187	94.0		
Exposure to other patients' suffering during hospitalization			0.376	0.673
**Psychosocial perceptions after discharge**				
Somatic symptoms after discharge			4.191	5.516
Perceived impact of being infected with COVID-19			10.155	9.186
Resource loss/gain			14.819	2.305
Resilience			7.518	1.854

As Table 2 showed, the significant background factors of PTSD symptoms included age, sex, marital status, having children, and occupation. In addition, ICU experience, exposure to/witnessing other patients' suffering during hospitalization, somatic symptoms after discharge, perceived impact of being infected with COVID-19, resource loss/gain, and resilience were significantly associated with PTSD symptoms (*p* < 0.05).

**Table 2:** Background factors, physical factors and psychological factors of PTSD symptoms.

**Variables**	**β**	**95%CI**	***P***	***F*(df)**	***R* ^2^**
Age	0.18	0.01, 0.10	0.008	7.348 (1/197)	0.03
Sex				10.61 (1/197)	0.04
Male	Ref				
Female	0.21	0.83, 3.67	0.002		
Relationship status				15.22 (1/197)	0.06
Currently single	Ref				
Married/cohabited with a partner	0.25	1.44, 4.55	<0.001		
Having children				11.80 (1/197)	0.07
Yes	Ref				
No	−0.27	−4.97, −1.75	<0.001		
Highest education attained				0.06 (2/196)	0.01
Junior high or below	Ref				
Senior high	0.07	−1.11, 2.72	0.41		
College and above	0.05	−1.21, 2.26	0.55		
Permanent resident of the city				0.03 (1/197)	0.00
Yes	Ref				
No	0.02	−1.36, 1.77	0.79		
Monthly income (RMB)					
<6,000	Ref			0.05 (2/196)	0.01
6,000 or above	−0.09	−2.91, 0.75	0.25		
No fixed income	−0.04	−2.16, 1.28	0.61		
Employment status				7.20 (4/194)	0.09
Full-time employment	−0.22	−4.24, −0.61	0.009		
Free-lanced	−0.03	−2.74, 1.78	0.68		
Students	−0.31	−7.14, −2.47	<0.001		
Unemployed	−0.12	−4.49, 0.49	0.12		
Retired	Ref				
Clinical classification of COVID-19 at entry				0.06 (4/194)	0.01
Asymptomatic	−0.06	−6.20, 2.90	0.476		
Mild	−0.07	−3.67, 1.86	0.520		
Common	0.15	−0.91, 4.12	0.210		
Severe	0.13	−0.96, 5.26	0.175		
Critically severe	Ref				
ICU experience				10.88 (1/197)	0.06
No	Ref				
Yes	0.23	3.43, 12.74	0.001		
Invasive assisted ventilation				0.26 (1/197)	0.00
No	Ref				
Yes	0.04	−2.81, 5.31	0.550		
Hormone therapy				0.79 (1/197)	0.00
No	Ref				
Yes	0.07	−1.07, 3.50	0.296		
Serious complications				0.07 (1/197)	0.00
No	Ref				
Yes	0.03	−2.66, 3.89	0.711		
Length of stay (days)	−0.04	−0.06, 0.03	0.538	0.64 (1/197)	0.00
Sequelae of COVID-19 before discharge				0.76 (1/197)	0.00
No	Ref				
Yes	−0.05	−4.38, 1.89	0.435		
Somatic symptoms after discharge	0.52	0.39, 0.62	<0.001	67.83 (1/197)	0.26
Exposure to other patients' suffering during hospitalization	0.23	0.74, 2.84	<0.001	16.46 (1/197)	0.08
Perceived impact of being infected with COVID-19	0.52	0.23, 0.37	<0.001	66.55 (1/197)	0.25
Resource loss/gain	−0.56	−1.59, −1.06	<0.001	55.63 (1/197)	0.22
Resilience	−0.38	−1.45, −0.73	<0.001	32.63 (1/197)	0.14

**Figure 1:**
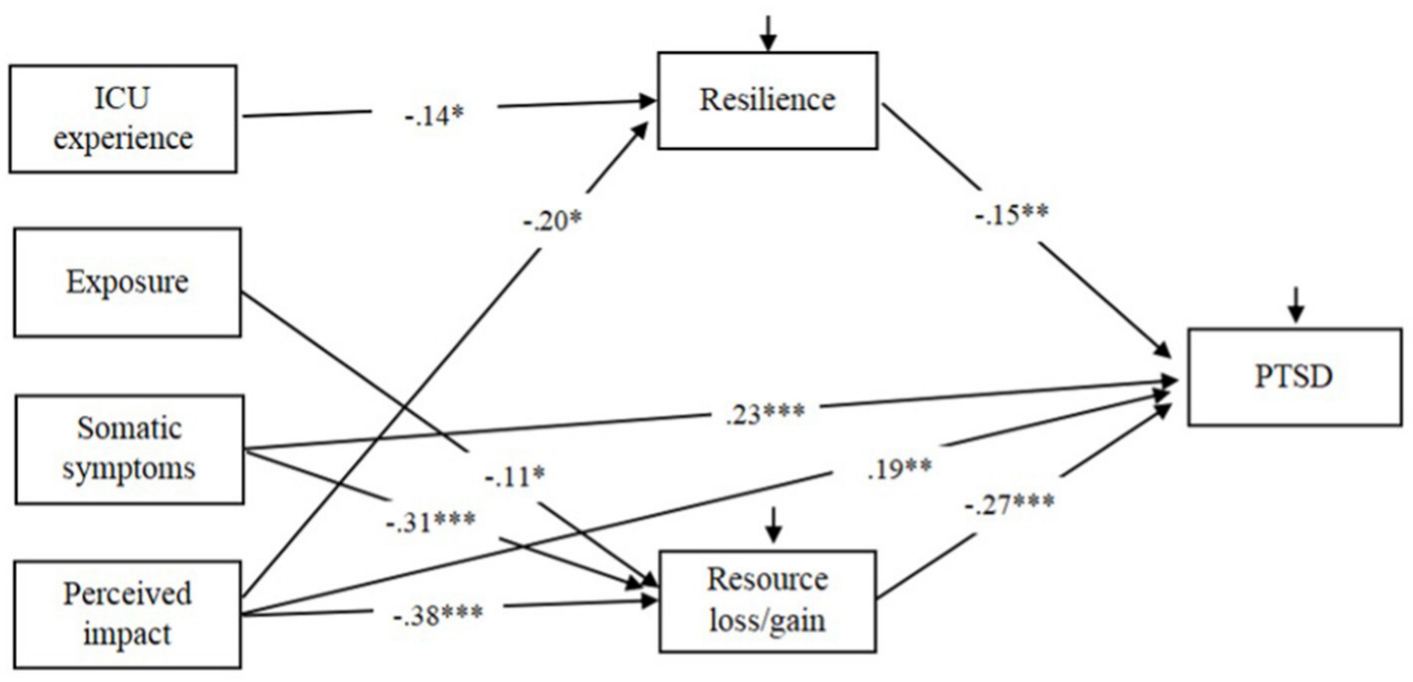
Proposed mediation model of PTSD symptoms with standardized path coefficients. The non-significant path and covariance between resilience and resource loss/gain were not showed for simplicity reasons (*p < 0.05, **p < 0.01, ***p < 0.001).

Figure 1 presented the proposed statistical mediation model of PTSD symptoms. The model showed good model fit, χ^2^(df) = 17.286 (5), *p* = 0.004, CFI = 0.962, NNFI = 0.951, RMSEA = 0.077. Perceived impact, somatic symptoms, and exposure to other patients' suffering were significantly negatively associated with gain of resources. In addition, perceived impact and ICU experience were significantly negatively associated with resilience. In turn, both resource gain and resilience were negatively associated with PTSD symptoms. High levels of perceived impact (*B* = 0.10, *β* = 0.10, 95%CI = 0.03, 0.18) and somatic symptoms (*B* = 0.08, β = 0.15, 95%CI = 0.08, 0.23) were indirectly associated with high levels of PTSD symptoms. Specifically, high perceived impact was indirectly associated with high PTSD symptoms through both reduced resource gain (*B* = 0.06, β = 0.10, *p* < 0.05) and resilience (*B* = 0.03, β = 0.04, *p* < 0.05), while somatic symptoms were indirectly associated with PTSD symptoms through resource loss/gain (*B* = 0.08, β = 0.08, *p* < 0.05). There were significant direct paths between high perceived impact and somatic symptoms and high PTSD symptoms. Thus, the partial statistical mediation effects of resource gain and resilience were demonstrated.

## Discussion

The results suggested several potential predictors, including socio-demographic, hospitalization and post-hospitalization variables, significantly associated with PTSD symptoms among COVID-19 survivors at 6 months after discharge. The results also illustrated the interrelationships among hospitalization variables, post-hospitalization variables, PTSD symptoms and the potential mediators based on the COR framework. The hypotheses were generally supported by the results.

The prevalence of probable PTSD at 6 months after discharge was less than that in other studies in which PTSD was measured during quarantine period or at 1 or 2 months after discharge (5–7). This seems consistent with the assertion that PTSD symptoms are often high among individuals hospitalized due to COVID-19, which may be due to the overlap between PTSD symptoms and acute illness (i.e., difficulties sleeping, feeling cut off from others, and difficultly concentrating), and may diminish after discharge (7). Notwithstanding, 7% of probable PTSD at 6 months after discharge indicate a great need for mental health care for this population. Follow-up studies should be conducted to estimate mental health care needs in COVID-19 survivors who are at high risk of PTSD.

Consistent with recent studies (5, 7), older, retired, non-single, or female survivors and those having children experienced more PTSD symptoms than their counterparts. These groups may be more vulnerable regarding their physical and socioeconomic status, and thus traumatic stress has more influence on them. Attention from health care providers should be warranted and more mental health promotion efforts should take place (44). ICU experience, witnessing other patients' suffering during hospitalization, somatic symptoms after discharge, and perceived impact of being infected with COVID-19 after discharge were significantly associated with PTSD symptoms. Such association may suggest that COVID-19 can have a long-term effect on survivors' well-being and quality of life even 6 months after their recovery and discharge. It also suggests that both hospitalization experience and post-hospitalization experience can play as continuous sources of stress which can fuel and prolong PTSD symptoms. Consistently, a recent study in Wuhan, China, also found that severity of disease and somatic symptoms after discharge were significant risk factors for PTSD among COVID-19 survivors (7). However, two studies in other cultures did not found such positive correlations (6, 8). Whether cultures play a role in the inconsistent findings is worth exploring in future studies (e.g., whether hospitalization experiences were different across cultures or people in some cultures are more sensitive to collective trauma). The two psychological factors, perceived resource loss/gain and resilience, were significantly associated with PTSD symptoms, suggesting that survivors who perceived more resource loss related to daily life functions and interpersonal relations, and reduced resilience experienced more PTSD symptoms. This is the first study testing these psychological correlates of PTSD among COVID-19 survivors, and the results are consistent with non-COVID-19 studies (25, 45). Other psychological factors that were not included in this study may also be important and can be tested in future work. For example, the fear of a new pandemic, worries amplified by media reporting, and uncertainty about the condition may aggravate PTSD symptoms among survivors. In addition, unlike other traumatic events (e.g., earthquake), COVID-19 survivors are, to this day, facing other problems related to COVID-19 such as lockdowns, business closing and unemployment, and thus may likely suffer from mental health problems.

Furthermore, the results support the statistical mediation roles of perceived resource loss/gain and resilience in explaining the potential effects of experience of hospitalization and post-hospitalization on PTSD symptoms. Specifically, perceived resource loss/gain fully mediated the association between witnessing other patients' suffering during hospitalization and PTSD symptoms as the indirect association *via* the mediator was statistically significant but not the direct association; perceived resource loss/gain partially mediated the associations between somatic symptoms/perceived impact of being infected with COVID-19 after discharge and PTSD symptoms as both the indirect and direct associations were statistically significant. Witnessing other patients' suffering might particularly reduce ones' personal resources through poor emotional status and quality of life, while somatic symptoms and perceived impact might affect ones' perceived resource loss in personal (e.g., sleep quality), social (e.g., relationship quality) and financial (e.g., job loss) aspects. Resilience was a full mediator of the association between ICU experience and PTSD symptoms as the indirect association *via* the mediator was statistically significant but not the direct association; it was also a partial mediator of the association between perceived impact and PTSD symptoms as both the indirect and direct associations were statistically significant. Being treated in ICU could be an extremely stressful event that might harm survivors' sense of mastery and psychological resources (12–14). These resources played critical roles in affecting survivors' stress coping and recovery from PTSD. In general, the relationship between in-hospital exposure to others' suffering and resource loss/gain and ICU experience to resilience are relatively weaker than those for post-hospital somatic symptoms and perceived impact. It may suggest that the lasting effects may be more important in contributing to PTSD symptoms than the acute in-hospital effects. The results of partial mediation and full mediation effects also seem to suggest that experience during hospitalization would not directly lead to PTSD at 6 months after discharge but would indirectly increase PTSD through perceived reduced psycho-social and personal resources, while experience after discharge had both direct and indirect effects on PTSD through reductions in such resources. Thus, experience after discharge might have more direct effects on PTSD than experience during hospitalization. It may echo the assertion in a recent study that PTSD symptoms from COVID-19 are high among hospitalized individuals, which may be due to the overlap between PTSD symptoms and acute illness (i.e., difficulties sleeping, feeling cut off from others, and difficultly concentrating), and diminish substantially after discharge (7).

Theoretically, the acceptable model fit and statistically significant mediation effects provide evidence for applying the COR theory (21) to understand how COVID-19-related experiences (e.g., hospitalization and post-hospitalization) may induce and prolong PTSD symptoms after discharge. From the COR perspective, people strive to develop, maintain or restore important resources in traumatic events, and a loss of these resources plays a critical role in developing PTSD (21). The COR theory has been applied in understanding mental health issues of survivors under other types of adversities (e.g., natural disasters and negative life events). To our knowledge, this is the first study that has extended and applied this theory to explain the development and maintenance of PTSD symptoms among COVID-19 survivors. Namely, the post-hospitalization experiences (somatic symptoms and perceived impacts on daily life, work, and socializing) rather than hospitalization experiences were highlighted as potential key sources of perceived resource losses which, in turn, might lead to PTSD. Other potentially important negative experiences and personal/interpersonal resources, such as physical pain, information deficiency, and loneliness due to COVID-19 infection, may also increase their PTSD symptoms and should be explored in future work.

Practically, the findings suggest some directions regarding the interventions for mental health promotion among COVID-19 survivors. Although adverse experiences during traumatic events are inevitable, the identified psychosocial status (e.g., resilience and interpersonal resources) can be modified through and the related negative consequences can be buffered by interventions. For example, some psychological interventions (e.g., the family-focused resilience enhancement program and the READY program) have been demonstrated to enhance resilience effectively after traumatic events (46). Timely social support from local governments and important others (e.g., family members and friends) of the survivors may help to buffer their perception of resource loss and facilitate their development of post-traumatic growth (47).

### Limitations

Our study has several limitations. First, this study used a cross-sectional design which limits causal inference. Although this study defined the mediators and outcome based on the theoretical model of COR, it is worth noting that the relationships between resilience/resource loss and PTSD might be reciprocal. In other words, individuals with resource losses would likely develop PTSD, while those with PTSD tend to perceive or experience actual resource losses. Thus, follow-up longitudinal studies are warranted to better understand their dynamic relationships and monitor to what extent hospitalization experience affects PTSD in the long run. Second, the study conveniently selected the hospitals where participants were recruited, making it potentially susceptible to sample bias. Only a few participants had ICU or ventilator treatment or had serious complications. Therefore, our results may not be generalizable to those more serious cases. This may partially explain the low probable PTSD estimate. Third, most measures (except the hospitalization records) were self-reported and might induce recall bias. In addition, the measures of perceived impact of being infected with COVID-19 and resource loss/gain were self-constructed by the research team based on previous studies. Although they showed acceptable reliability, it is worth noting that they have not been well-validated. Resilience was measured by the 2-item short-version scale. The long-version resilience scale including multiple dimensions may measure resilience in a more comprehensive way. Future studies should validate the findings using more robust measures. In addition, a limitation of the resource loss/gain measure and the PTSD measure is that they may be confounded by the effects of overall exposure to the pandemic (and related traumas and losses) vs. the specific effects of COVID-19 infection. Future studies may further specify the sources of resource loss/gain and PTSD in the measures or compare the levels of these factors with those among people without COVID-19 infection. Fourth, we did not have the records of mental health status before hospitalization of the participants. This is a significant limitation, as prior depression, anxiety, or PTSD due to other trauma exposure would be significant risk factors for symptom recurrence during and after hospitalization. Future studies should collect and control for this information to better understand the burden due to COVID-19 infection. In addition, we were not able to access refusers' medical records without their approval or collect other information from the refusers. Therefore, we were not able to compare the characteristics between refusers and participants. Fifth, due to the small sample size of the whole sample and those with probable PTSD, we did not use the binary variable of PTSD in our analyses. Last but not least, caution of interpreting the prevalence of PTSD should be made as the tool is not a diagnostic instrument. The cutoff has not been clinically validated in Chinese populations. Future studies need to validate the cutoff of the PTSD scale and investigated the prevalence of PTSD in a larger and representative sample.

## Conclusion

The effects of COVID-19 on survivors can extend beyond the physical affliction and continue even after recovery and hospital discharge. The significant potential factors relating to PTSD symptoms included socio-demographic status (age, sex, marital status, having children, and occupation), hospitalization experiences (ICU experience and witnessing other patients' suffering during hospitalization), post-hospitalization experiences (somatic symptoms after discharge and perceived impact of being infected with COVID-19), and psychological status (resource loss/gain and resilience). In addition, the psychological status served as significant mediators in explaining the associations between experiences of hospitalization and post-hospitalization and PTSD symptoms. Such psychosocial mechanisms are modifiable through psycho-social interventions. Efforts to enhance survivors' resilience and resource gain are warranted to reduce and prevent PTSD.

## Data Availability Statement

The raw data supporting the conclusions of this article will be made available by the authors, without undue reservation.

## Ethics Statement

The studies involving human participants were reviewed and approved by Sun Yat-sen University (Shenzhen). Written informed consent for participation was not required for this study in accordance with the national legislation and the institutional requirements. Each participant's informed consent was confirmed before the interview.

## Author Contributions

HZo, BW, and LF conceived the idea. XY, YF, PChan, and ZW organized a questionnaire. HZo, FX, SY, JYu, Y-QC, XX, BW, and LF designed the investigation. BW, LF, YHu, DL, XX, NJ, WZ, HX, ZX, PChen, JH, HZh, HT, DH, ZH, XM, YHa, LC, and JYa carried out this investigation. XY and BW prepared the manuscript with ZW and HZo. XY, BW, and ZW critically reviewed the manuscript. All authors contributed to the article and approved the submitted version.

## Funding

This study was supported by the Natural Science Foundation of China Excellent Young Scientists Fund (82022064), Natural Science Foundation of China International/Regional Research Collaboration Project (72061137001), Natural Science Foundation of China Young Scientist Fund (81703278), the Australian National Health and Medical Research Commission (NHMRC) Early Career Fellowship (APP1092621), the National Science and Technology Major Project of China (2018ZX10721102), the Sanming Project of Medicine in Shenzhen (SZSM201811071), the High Level Project of Medicine in Longhua, Shenzhen (HLPM201907020105), the National Key Research and Development Program of China (2020YFC0840900), and Guangxi Medical and Health Appropriate Technology Development and Application Project (S2020124). All funding parties did not have any role in the design of the study or in the explanation of the data.

## Conflict of Interest

The authors declare that the research was conducted in the absence of any commercial or financial relationships that could be construed as a potential conflict of interest.

## Publisher's Note

All claims expressed in this article are solely those of the authors and do not necessarily represent those of their affiliated organizations, or those of the publisher, the editors and the reviewers. Any product that may be evaluated in this article, or claim that may be made by its manufacturer, is not guaranteed or endorsed by the publisher.

## References

[1] Shalev A Liberzon I Marmar C. Posttraumatic stress disorder. New Engl J Med Rev. (2017) 376:2459–69. 10.1056/NEJMra1612499 28636846

[2] Varela VS Ng A Mauch P Recklitis CJ. Posttraumatic stress disorder (PTSD) in survivors of Hodgkin's lymphoma: prevalence of PTSD and partial PTSD compared with sibling controls. Psychooncology. (2013) 22:434–40. 10.1002/pon.2109 22162210 PMC3908687

[3] Lee SH Shin HS Park HY Kim JL Lee JJ Lee H. Depression as a mediator of chronic fatigue and post-traumatic stress symptoms in middle east respiratory syndrome survivors. Psychiatry Investig. (2019) 16:59–64. 10.30773/pi.2018.10.22.3 30605995 PMC6354037

[4] Mak IWC Chu CM Pan PC Yiu MGC Ho SC Chan VL. Risk factors for chronic post-traumatic stress disorder (PTSD) in SARS survivors. General Hospital Psychiatry. (2010) 32:590–8. 10.1016/j.genhosppsych.2010.07.007 21112450 PMC7132390

[5] Cai X Hu X Ekumi IO Wang J An Y Li Z. Psychological distress and its correlates among COVID-19 survivors during early convalescence across age groups. Am J Geriatr Psychiatry. (2020) 28:1030–9. 10.1016/j.jagp.2020.07.003 32753338 PMC7347493

[6] Mazza MG De Lorenzo R Conte C Poletti S Vai B Bollettini I. Anxiety and depression in COVID-19 survivors: role of inflammatory and clinical predictors. Brain Behav Immun. (2020) 89:594–600. 10.1016/j.bbi.2020.07.037 32738287 PMC7390748

[7] Liu D Baumeister RF Veilleux JC Chen C Liu W Yue Y. Risk factors associated with mental illness in hospital discharged patients infected with COVID-19 in Wuhan, China. Psychiatry Res. (2020) 292:113297. 10.1016/j.psychres.2020.113297 32707218 PMC7355324

[8] De Lorenzo R Conte C Lanzani C Benedetti F Roveri L Mazza MG. Residual clinical damage after COVID-19: a retrospective and prospective observational cohort study. PLoS ONE. (2020) 15:e0239570. 10.1371/journal.pone.0239570 33052920 PMC7556454

[9] Tarsitani L Vassalini P Koukopoulos A Borrazzo C Alessi F Di Nicolantonio C. Post-traumatic stress disorder among COVID-19 survivors at 3-month follow-up after hospital discharge. J Gen Intern Med. (2021) 36:1702–7. 10.1007/s11606-021-06731-7 33782888 PMC8007055

[10] Kaseda ET Levine AJ. Post-traumatic stress disorder: a differential diagnostic consideration for COVID-19 survivors. Clin Neuropsychol. (2020) 34:1498–514. 10.1080/13854046.2020.1811894 32847484

[11] Xie J Tong Z Guan X Du B Qiu H Slutsky AS. Critical care crisis and some recommendations during the COVID-19 epidemic in China. Intens Care Med. (2020) 46:837–40. 10.1007/s00134-020-05979-7 32123994 PMC7080165

[12] Cuthbertson BH Hull A Strachan M Scott J. Post-traumatic stress disorder after critical illness requiring general intensive care. Intensive Care Med. (2004) 30:450–5. 10.1007/s00134-003-2004-8 12961065

[13] Griffiths J Fortune G Barber V Young JD. The prevalence of post traumatic stress disorder in survivors of ICU treatment: a systematic review. Intensive Care Med. (2007) 33:1506–18. 10.1007/s00134-007-0730-z 17558490

[14] Taylor AK Fothergill C Krige A Chew-Graham CA Patel S. Identification of post-traumatic stress disorder following ICU. Br J Gen Pract. (2019) 69:154–5. 10.3399/bjgp19X701765 30819760 PMC6400613

[15] Chamberlain SR Grant JE Trender W Hellyer P Hampshire A. Post-traumatic stress disorder symptoms in COVID-19 survivors: online population survey. BJPsych Open. (2021) 7:1–4. 10.1192/bjo.2021.3 33557964 PMC7873456

[16] Shaw RJ Harvey JE Bernard R Gunary R Tiley M Steiner H. Comparison of short-term psychological outcomes of respiratory failure treated by either invasive or non-invasive ventilation. Psychosomatics. (2009) 50:586–91. 10.1016/S0033-3182(09)70860-6 19996229

[17] Twigg E Humphris G Jones C Bramwell R Griffiths RD. Use of a screening questionnaire for post-traumatic stress disorder (PTSD) on a sample of UK ICU patients. Acta Anaesthesiol Scand. (2008) 52:202–8. 10.1111/j.1399-6576.2007.01531.x 18005373

[18] Gosselin E Gélinas C Bourgault P Lavoie S. Intervention for patients intubated and conscious to decrease peritraumatic distress (IPIC-PTD) – acceptability and feasibility. Sci Nurs Health Pract. (2018) 1:1019. 10.31770/2561-7516.1019

[19] Girard TD Shintani AK Jackson JC Gordon SM Pun BT Henderson MS. Risk factors for post-traumatic stress disorder symptoms following critical illness requiring mechanical ventilation: a prospective cohort study. Critical Care. (2007) 11:1–8. 10.1186/cc5708 17316452 PMC2151865

[20] Sheehy LM. Considerations for postacute rehabilitation for survivors of COVID-19. JMIR Public Health Surv. (2020) 6:1–8. 10.2196/19462 32369030 PMC7212817

[21] Hobfoll SE. Conservation of resources: a new attempt at conceptualizing stress. Am Psychol. (1989) 44:513–24. 10.1037/0003-066X.44.3.513 2648906

[22] Kakodkar P Nagham K Baig MN. A comprehensive literature review on the clinical presentation, and management of the pandemic coronavirus disease 2019 (COVID-19). Cureus. (2020) 12:e7560. 10.7759/cureus.7560 32269893 PMC7138423

[23] Asimakopoulou E Madianos M. The prevalence of major depression-PTSD comorbidity among ICU survivors in five general hospitals of athens: a cross-sectional study. Issues Ment Health Nurs. (2014) 35:954–63. 10.3109/01612840.2014.924609 25325150

[24] Hobfoll SE Vinokur AD Pierce PF Lewandowski-Romps L. The combined stress of family life, work, and war in Air Force men and women: a test of conservation of resources theory. Int J Stress Manag. (2012) 19:217–37. 10.1037/a0029247

[25] Alvaro C Lyons RF Warner G Hobfoll SE Martens PJ Labonté R. Conservation of resources theory and research use in health systems. Implement Sci. (2010) 5:79. 10.1186/1748-5908-5-79 20961445 PMC2978118

[26] Diener E Oishi S Lucas RE. Personality, culture, and subjective well-being: emotional and cognitive evaluations of life. Ann Rev Psychol. (2003) 54:403–25. 10.1146/annurev.psych.54.101601.145056 12172000

[27] Lu L Chou CY Zeng YLY Cooper CL. Personal and social resources in coping with long hours of the Chinese work condition: the dual roles of detachment and social motivation. Int J Hum Resource Manag. (2020) 1–35. 10.1080/09585192.2020.1779778

[28] Benight CC Ironson G Durham RL. Psychometric properties of a hurricane coping self-efficacy measure. J Trauma Stress. (1999) 12:379–86. 10.1023/A:1024792913301 10378175

[29] Freedy JR Saladin ME Kilpatrick DG Resnick HS Saunders BE. Understanding acute psychological distress following natural disaster. J Trauma Stress. (1994) 7:257–73. 10.1002/jts.2490070207 8012746

[30] Taylor HO Taylor RJ Nguyen AW Chatters L. Social isolation, depression, and psychological distress among older adults. J Aging Health. (2018) 30:229–46. 10.1177/0898264316673511 28553785 PMC5449253

[31] Hall KS Kusunoki Y Gatny H Barber J. Social discrimination, stress, and risk of unintended pregnancy among young women. J Adolesc Health. (2015) 56:330–7. 10.1016/j.jadohealth.2014.11.008 25586228 PMC4339533

[32] Bo H-X Li W Yang Y Wang Y Zhang Q Cheung T. Posttraumatic stress symptoms and attitude toward crisis mental health services among clinically stable patients with COVID-19 in China. Psychol Med. (2021) 51:1052–3. 10.1017/S0033291720000999 32216863 PMC7200846

[33] Wang B Fu L Ju N Xiao X Zou H. Interviews to better understand the burden of mental health disease among COVID-19 survivors: things to consider. J Affect Disord. (2021) 285:84–5. 10.1016/j.jad.2021.02.044 33636675 PMC7887447

[34] Kroenke K Spitzer RL Williams JBW. The PHQ-15: validity of a new measure for evaluating the severity of somatic symptoms. Psychosom Med. (2002) 64:258–66. 10.1097/00006842-200203000-00008 11914441

[35] Liu G Clark MR Eaton WW. Structural factor analyses for medically unexplained somatic symptoms of somatization disorder in the Epidemiologic Catchment Area Study. Psychol Med. (1997) 27:617–26. 10.1017/S0033291797004844 9153682

[36] Hobfoll SE. Traumatic stress: a theory based on rapid loss of resources. Anxiety Research. (1991) 4:187–97. 10.1080/0891777910824877317453549

[37] Hansen M Andersen TE Armour C Palic S Mackrill T. PTSD-8: a short PTSD inventory. Clin Pract Epidemiol Mental Health. (2010) 6:101–8. 10.2174/1745017901006010101 21253461 PMC3023946

[38] Andersen TE Hansen M Ravn SL Seehuus R Nielsen M Vaegter HB. Validation of the PTSD-8 scale in chronic pain patients. Pain Med (United States). (2018) 19:1365–72. 10.1093/pm/pnx166 29016902

[39] Khan S Haque S. Trauma, mental health, and everyday functioning among Rohingya refugee people living in short- and long-term resettlements. Soc Psychiatry Psychiatric Epidemiol. (2021) 56:497–512. 10.1007/s00127-020-01962-1 33015727

[40] O'connor K Seager J. Displacement, violence, and mental health: evidence from rohingya adolescents in cox's bazar, Bangladesh. Int J Environ Res Public Health. (2021) 18:5318. 10.3390/ijerph18105318 34067724 PMC8156348

[41] Arditte Hall KA DeLane SE Anderson GM Lago TR Shor R Wang W. Plasma gamma-aminobutyric acid (GABA) levels and posttraumatic stress disorder symptoms in trauma-exposed women: a preliminary report. Psychopharmacology. (2021) 238:1541–52. 10.1007/s00213-021-05785-z 33620549

[42] Yang X Yip BHK Mak ADP Zhang D Lee EKP Wong SYS. The differential effects of social media on depressive symptoms and suicidal ideation among the younger and older adult population in hong kong during the covid-19 pandemic: population-based cross-sectional survey study. JMIR Public Health Surveillance. (2021) 7:1–15. 10.2196/24623 33835937 PMC8153033

[43] Yang X Yip BHK Lee EKP Zhang D Wong SYS. The relationship between technology use and problem technology use and potential psychosocial mechanisms: population-based telephone survey in community adults during COVID-19. Front Psychol. (2021) 12:e696271. 10.3389/fpsyg.2021.696271 34434146 PMC8381748

[44] Boscarino REA Boscarino JA. Predictors of PTSD and delayed PTSD after disaster: the impact of exposure and psychosocial resources. J Nerv Ment Dis. (2006) 194:485–93. 10.1097/01.nmd.0000228503.95503.e9 16840844 PMC2712250

[45] Zang Y Gallagher T McLean CP Tannahill HS Yarvis JS Foa EB. The impact of social support, unit cohesion, and trait resilience on PTSD in treatment-seeking military personnel with PTSD: the role of posttraumatic cognitions. J Psychiatr Res. (2017) 86:18–25. 10.1016/j.jpsychires.2016.11.005 27886636

[46] Burton NW Pakenham KI Brown WJ. Feasibility and effectiveness of psychosocial resilience training: a pilot study of the READY program. Psychol Health Med. (2010) 15:266–77. 10.1080/13548501003758710 20480432

[47] Yang X Wang Q Wang X Mo PKH Wang Z Lau JTF. Direct and indirect associations between interpersonal resources and posttraumatic growth through resilience among women living with HIV in China. AIDS Behav. (2020) 24:1687–700. 10.1007/s10461-019-02694-3 31624976

